# Expression of Wild-Type Rp1 Protein in *Rp1* Knock-in Mice Rescues the Retinal Degeneration Phenotype

**DOI:** 10.1371/journal.pone.0043251

**Published:** 2012-08-21

**Authors:** Qin Liu, Rob W. J. Collin, Frans P. M. Cremers, Anneke I. den Hollander, L. Ingeborgh van den Born, Eric A. Pierce

**Affiliations:** 1 Berman-Gund Laboratory for the Study of Retinal Degenerations, Ocular Genomics Institute, Department of Ophthalmology, Massachusetts Eye and Ear Infirmary, Boston, Massachusetts, United States of America; 2 Department of Human Genetics, Radboud University Nijmegen Medical Centre, Nijmegen, The Netherlands; 3 Department of Ophthalmology, Radboud University Nijmegen Medical Centre, Nijmegen, The Netherlands; 4 Nijmegen Centre for Molecular Life Sciences, Radboud University Nijmegen Medical Centre, Nijmegen, The Netherlands; 5 The Rotterdam Eye Hospital, Rotterdam, The Netherlands; Innsbruck Medical University, Austria

## Abstract

Mutations in the retinitis pigmentosa 1 (*RP1*) gene are a common cause of autosomal dominant retinitis pigmentosa (adRP), and have also been found to cause autosomal recessive RP (arRP) in a few families. The 33 dominant mutations and 6 recessive *RP1* mutations identified to date are all nonsense or frameshift mutations, and almost exclusively (38 out of 39) are located in the 4^th^ and final exon of *RP1*. To better understand the underlying disease mechanisms of and help develop therapeutic strategies for RP1 disease, we performed a series of human genetic and animal studies using gene targeted and transgenic mice. Here we report that a frameshift mutation in the 3^rd^ exon of *RP1* (c.686delC; p.P229QfsX35) found in a patient with recessive *RP1* disease causes RP in the homozygous state, whereas the heterozygous carriers are unaffected, confirming that haploinsufficiency is not the causative mechanism for *RP1* disease. We then generated *Rp1* knock-in mice with a nonsense Q662X mutation in exon 4, as well as *Rp1* transgenic mice carrying a wild-type BAC *Rp1* transgene. The *Rp1*-Q662X allele produces a truncated Rp1 protein, and homozygous *Rp1*-Q662X mice experience a progressive photoreceptor degeneration characterized disorganization of photoreceptor outer segments. This phenotype could be prevented by expression of a normal amount of Rp1 protein from the BAC transgene without removal of the mutant Rp1-Q662X protein. Over-expression of Rp1 protein in additional BAC *Rp1* transgenic lines resulted in retinal degeneration. These findings suggest that the truncated Rp1-Q662X protein does not exert a toxic gain-of-function effect. These results also imply that in principle gene augmentation therapy could be beneficial for both recessive and dominant *RP1* patients, but the levels of RP1 protein delivered for therapy will have to be carefully controlled.

## Introduction

Recent reports of the success of Phase I clinical trials of gene therapy for Leber congenital amaurosis (LCA) and X-linked adrenoleukodystrophy (ALD) indicate that we are entering an era of genetic therapies, at least for some forms of genetic disease [1]–[8]. Since these initial successes are based on gene augmentation therapy, it has become increasingly important to determine the mechanisms by which mutations identified to cause inherited disorders such as LCA and retinitis pigmentosa (RP) exert their pathologic effects. Mutations can cause disease via a protein’s loss-of-function, gain-of-function or dominant-negative activity [9], [10]. Disease caused by loss-of-function or dominant-negative mutations, in which the mutant protein competes with the wild-type protein and blocks its full function, are potentially amenable to treatment with gene augmentation therapy. In contrast, treatment of dominant disorders in which the mutant protein acquires a novel toxic function (gain-of-function mutations) will require removal or suppression of the mutant allele [9], [10]. Despite the importance of distinguishing between different function of mutations, mechanisms have been determined for only a limited number of dominantly inherited disorders [11]. This is especially true for inherited retinal degenerations (IRDs), one of the most genetically diverse group of inherited disorders [12]. IRDs result in blindness by causing dysfunction and ultimately death of the rod and cone photoreceptor cells of the retina [13], [14]. Since the first IRD genes, rhodopsin (*RHO*) and *CHM*, were discovered two decades ago [15], [16], over 180 disease genes have been identified, which account for approximately 50–60% of the genetic causes of disease in IRD patients [17]–[19]. Retinitis pigmentosa (RP) is the most common form of IRD, affecting 1∶1000 to 1∶4000 people worldwide [18], [20]. It is characterized by nyctalopia and visual field loss, followed by loss of central vision, eventually leading to blindness.

**Figure 1:**
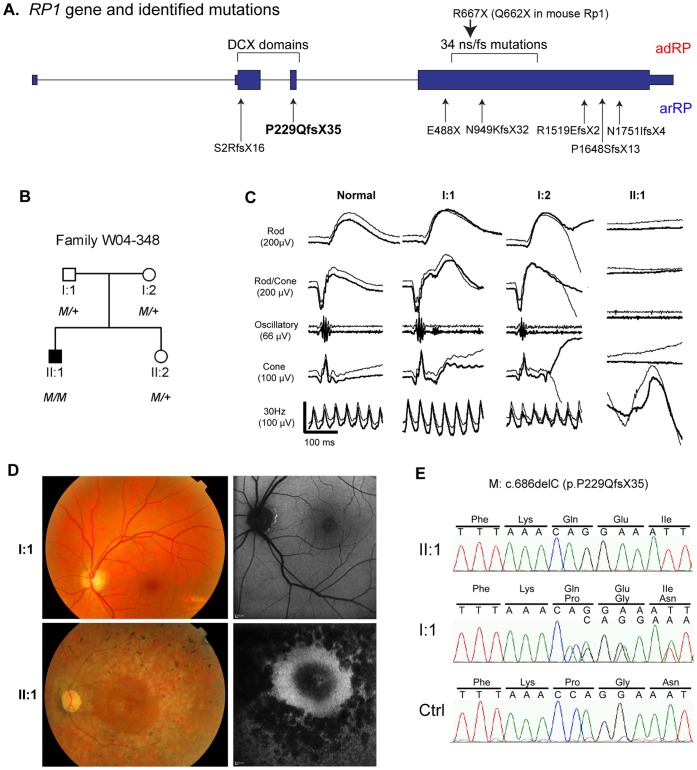
*RP1* Gene, Clinical and Sequence Data for Family W04-348. **A.** *RP1* gene and identified mutations. The gene structure of *RP1* is depicted, with the locations of mutations that cause adRP and arRP indicated; the mutations above the *RP1* gene structure cause dominant RP, labeled as adRP in red; whereas mutations below the *RP1* gene structure cause recessive RP, labeled as arRP in blue; frameshift mutation p.P229QfsX35 reported to cause arRP in this study is in bold. The portion of the gene that encodes that DCX domains is also indicated. The arrow on the top indicates the location of the R677X (human) and Q662X (mouse) mutations. **B.** Pedigree for family W04-348. The c.686delC, p.P229QfsX35 mutation is designated by *M*. **C.** ERG traces from a normal control, the patient’s parents (I-1, I-2 (age 57), and the affected patient (II-1). The five standard ISCEV recordings are shown, from the top including: scotopic rod responses, scotopic combined rod-cone responses, oscillatory potentials, photopic single flash and photopic 30Hz responses. The amplitude and time scales are indicated. The ERG responses of the patient’s parents show normal amplitudes and implicit times; the patient had no recordable rod or cone responses. The deflections shown in the 30 Hz recordings for the patient are due to motion artifact. The thicker traces are from the right eye, the thinner from the left eye. **D.** Fundus photos (left) and fundus autofluorescence images (right) of the affected patient II-1(age 30) and his father I-1 (age 60). The patient has typical findings of RP, with optic disc pallor, attenuation of the retinal blood vessels, RPE atrophy and bone spicule pigmentation outside the macula. As shown in the autofluorescence image, the RPE in the macular region is relatively preserved. In contrast, the father’s fundi are normal. **E.** Sequence traces showing the homozygous c.686delC mutation in patient II-1, carrier status of this mutation in the patient’s father I-1, and the wild-type sequence in an unaffected Dutch control. Note that the sequence trace of the mutant allele in individual I-1 is shifted slightly.

Mutations in the *RP1* gene are the second most common cause of autosomal dominant RP (adRP; 5.5%), and have also been found to cause autosomal recessive RP (arRP) [21]–[34]. Studies to date have shown that the RP1 protein (2156 aa, 240 kDa) is part of the axoneme of photoreceptor sensory cilia (PSC; also called outer segments) [35]. It is a photoreceptor-specific microtubule-associated protein (MAP) that is required for normal organization of membrane discs in the light-sensitive PSC of rod and cone cells. Microtubule binding domains in the N-terminal portion of RP1 mediate its interaction with the axoneme, and the C-terminal portion of the protein is hypothesized to interact with other proteins to help mediate the organization of outer segment discs [36]–[38]. To date, 33 mutations in *RP1* have been identified to cause adRP (Figure 1A). These are all nonsense or frameshift mutations clustered at the beginning of the 4^th^ and final exon of *RP1* (Figure 1A) [21]–[33]. Compared to the autosomal dominant form, only a small number of families with arRP due to mutations in *RP1* have been reported, including 4 homozygous mutations (c.1606insTGAA, c.2847delT, c.4703delA, and c.5400delA) and one pair of compound heterozygous mutations (c.5_6delGT and c.4941_4942insT) (Figure 1A)[34], [39]–[41]. These are also frameshift mutations, with 5 out of 6 located in the final exon as well. Since nonsense-mediated decay (NMD) is thought not to occur if the nonsense mutation is in the last exon, all those dominant and recessive mutations in the last exon of *RP1* are expected to produce stable transcripts, resulting in the production of truncated RP1 proteins that lack the C-terminal 1/3 rd to 2/3 rds of the full length RP1 protein in the photoreceptor cells of RP patients. Our previous studies on lymphoblasts of patients with *RP1* disease and gene targeted *Rp1*-tm1EAP mice suggest that the mutant *RP1* mRNAs with nonsense mutations in exon 4 are expected to escape NMD, but the hypothesis that premature termination codons in exon 4 lead to the production of truncated Rp1 proteins *in vivo* has not been tested empirically with a representative mutant allele [37].

To elucidate the molecular mechanisms of *RP1* mutations so that therapeutic strategies can be developed for *RP1* disease, we performed human genetic studies and experiments using several lines of *Rp1* gene targeted and transgenic mice. First, we evaluated the family of an adult patient with recessive RP due to a homozygous mutation in exon 3 of *RP1*, which is predicted to be null. The sibling and parents of this patient, who all carry a single mutant *RP1* allele, do not exhibit evidence of RP, confirming that functional hemizygosity of *RP1* does not cause disease [41]. Next, we generated and characterized *Rp1* knock-in mice with a Q662X nonsense point mutation in exon 4 of human *RP1*. As predicted, the mutant *Rp1*-Q662X allele produces a truncated Rp1 protein, and homozygous *Rp1*-Q662X experience photoreceptor degeneration. Third, we generated and characterized transgenic mice that produce a tagged version of the full-length Rp1 protein. Expression of normal levels of Rp1 protein from the transgene is well tolerated, but lines of transgenic mice that over-express the Rp1 protein experience retinal degeneration. Fourth, expression of wild-type Rp1 protein in homozygous *Rp1*-Q662X mice prevented photoreceptor degeneration. These results indicate that the truncated *Rp1*-Q662X protein does not exert a toxic gain-of-function effect in the retina, suggesting that dominant mutations in *RP1* do not cause disease via a gain-of-function mechanism.

## Results

### Haploinsufficiency of RP1 does not Cause Retinal Degeneration in Humans

As part of a large study to identify the genetic causes of RP in The Netherlands via homozygosity mapping [42], we found the disease-causing mutation for a Dutch patient with RP (Figure 1B–E). In this patient, whole-genome homozygosity mapping revealed a significant homozygous region of 8.3 Mb on chromosome 8, between rs1394468 and rs936616, in which the *RP1* gene resides. Mutation analysis of *RP1* in this patient revealed the homozygous 1-bp deletion in exon 3 (c.686delC) that is predicted to result in a frame-shift and premature termination of the protein (p.P229QfsX35; Figure 1A, E). Mutation analysis in the unaffected parents and sister of the patient revealed that these three individuals carried the mutation heterozygously (Figure 1B). This is the first mutation reported to date that causes arRP in the homozygous state, but is not located in the final exon.

The proband in this family (individual II-1, Figure 1) experienced nyctalopia from early childhood, and was diagnosed with RP at age 13. At that time, visual acuity was 0.6 in the right eye and 0.5 in the left eye, and electroretinogram (ERG) recordings were severely reduced with a rod-cone pattern. On recent examination at age 30, visual acuity was light perception in both eyes. The ERG was non-recordable (Figure 1C). Ophthalmoscopy revealed moderate pallor of the optic discs and attenuation of the retinal blood vessels (Figure 1D). The retinal pigment epithelium (RPE) in the posterior pole was relatively preserved, with subfoveal atrophy. Bone spicule pigmentation and RPE atrophy were observed in the retinal peripheries. In contrast, examination of the heterozygous parents (ages 60 and 57 respectively) and sister (age 28) did not reveal any abnormalities. None of these family members had nyctalopia, and ERG recordings showed normal retinal function for both parents (Figure 1C).

The observation that carriers of the c.686delC mutation do not have RP is consistent with a recent report describing a patient with arRP due to compound heterozygous frameshift mutations in *RP1*, c.5_6delGT (p.S2RfsX16, exon 2) and c.4941_4942insT (p.P1648SfsX13, exon 4). In that family, the carrier of the p.S2RfsX16 allele, which creates a premature termination codon in exon 2, did not show evidence of RP either [41]. Since the nonsense mutations, c.5_6delGT (p.S2RfsX16, exon 2) and c.686delC (p.P229QfsX35, exon 3), occur in the 2^nd^ or 3^rd^ rather than the final exon in these two families, they are expected to lead to NMD of the mutant mRNAs, and thus create null alleles [43]. These findings from two recessive *RP1* families suggest that haploinsufficiency of *RP1* does not cause retinal degeneration in humans.

### Generation and Characterization of Rp1-Q662X Knock-in Mice

**Figure 2:**
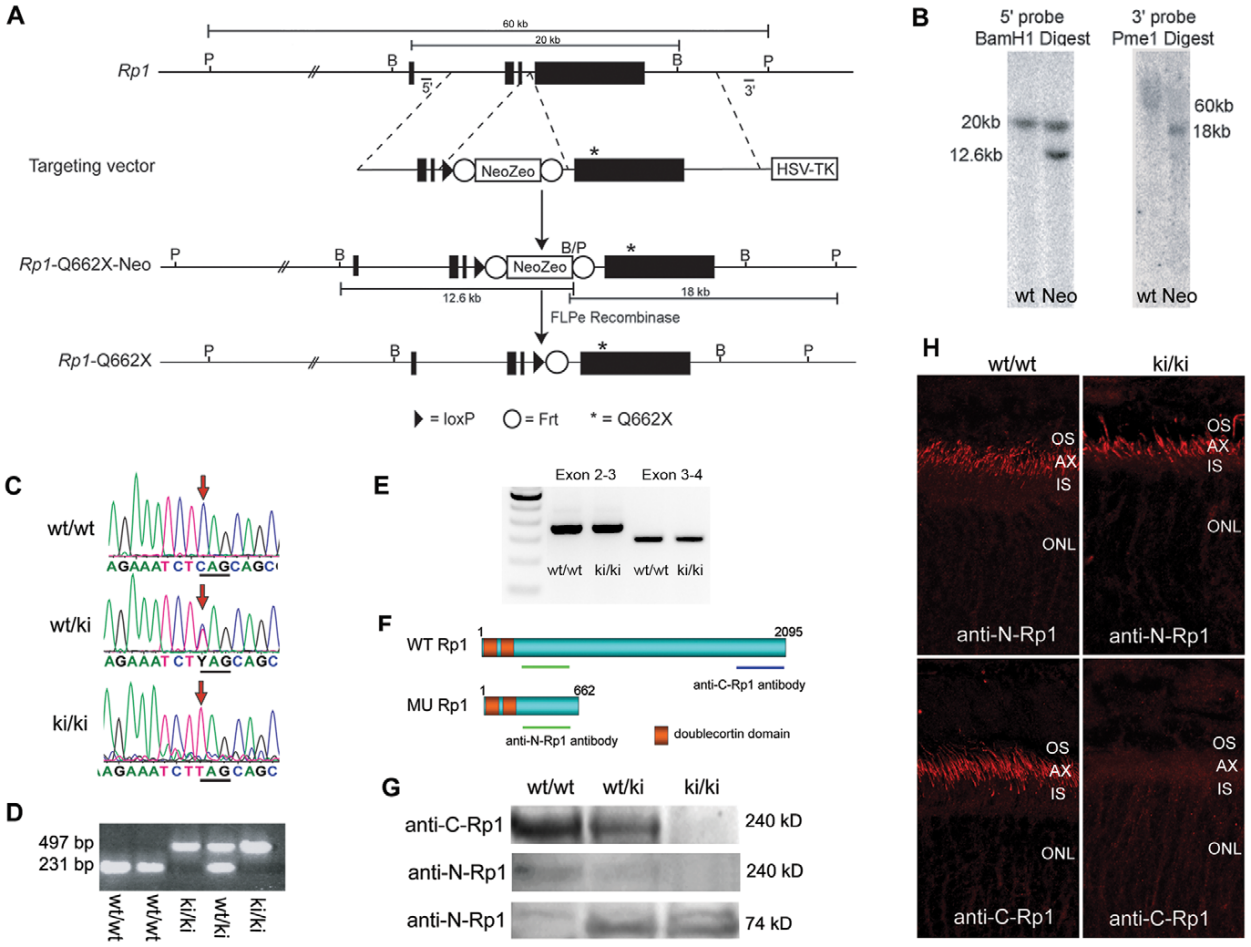
Generation and Characterization of *Rp1* ^Q662X/Q662X^ Knock-in Mice. A. Gene targeting strategy to introduce Q662X mutation into the endogenous *Rp1* locus. The gene targeting vector was produced by introducing the Neo-Zeo selection cassette and Q662X mutation into the *Rp1* gene in a BAC which contains 140 kb of mouse genomic DNA including the *Rp1* locus, using homologous recombination techniques. The gene targeting vector was then retrieved from the modified BAC, and used to transfect mouse ES cells. Correctly targeted ES cells were injected into blastocysts to generate *Rp1*-Neo-Q662X mice. Crosses to Flpe deleter mice were used to remove the Frt-flanked Neo-Zeo selection cassette and generate the final *Rp1*-Q662X allele. B, *Bam*HI restriction sites; P, *Pme*I restriction sites. **B.** Southern blots using the 5′ and 3′ probes indicated in A demonstrating correct targeting of the *Rp1* locus in mouse ES cells. The 12.6 and 18 kb bands in the 5′ and 3′ blots, respectively, indicate correct recombination to generate the *Rp1*-Neo-Q662X allele. The large size of the wild-type Pme1 restriction fragment (60 kb) in the 3′ blot makes it difficult to resolve this band with standard electrophoresis techniques. **C.** Sequence traces from PCR products amplified from wild-type, heterozygous (wt/ki) and homozygous (ki/ki) mice. **D.** Example PCR-based genotyping of *Rp1*-Q662X (ki) knock-in mice. Using PCR primers that amplify across the residual Frt-LoxP sites, the wild-type and ki alleles can be readily distinguished. **E.** RT-PCR of retinal RNA from mice of indicated genotypes showing the correct splicing of mutant Rp1-Q662X allele as that of wild type Rp1 allele, using primer sets bridging exon 2 to 3 and exon 3 to 4. **F.** Predicted proteins produced by the wild-type (wt) and knock-in (ki) *Rp1* alleles, showing locations of the anti-N-Rp1 and anti-C-Rp1 antibodies used in panels G and H. **G.** Western blot of retinal extracts from mice of the indicated genotypes showing production of the 74 kD truncated protein by the knock-in (ki) allele. Note that there is no full-length Rp1 protein detected in the retinas of the homozygous ki/ki mice by either the anti-N-Rp1 or anti-C-Rp1 antibodies. **H.** Immunofluorescence analysis of Rp1 proteins (red) produced in the retinas of wild-type (wt/wt) vs. homozygous knock-in (ki/ki) mice using the anti-N-Rp1 and anti-C-Rp1 antibodies indicated in panel E. Note that the truncated Rp1 protein produced in the retinas of the ki/ki mice locates correctly to the axonemes of PSC, just like the full-length protein in the wt/wt retinas. Note also the lack of full-length Rp1 protein production in the retinas of the ki/ki mice (AX, axoneme; IS, inner segment; ONL, outer nuclear layer; OS, outer segment).

We next focused on determining the possible disease mechanism for mutations identified in the final exon of human *RP1*. We hypothesized that *RP1* transcripts with stop mutations in the 4^th^ exon can escape NMD and produce truncated proteins, as predicted from previous studies [37]. To empirically address this question, we generated knock-in mice with a Q662X nonsense point mutation in the *Rp1* gene to represent those mutations that create premature termination codons in exon 4 of human *RP1* [21]–[33]. The *Rp1*-Q662X targeting vector was generated from a mouse bacterial artificial chromosome (BAC) clone by introducing the Q662X mutation along with a Frt-flanked neomycin selection cassette into the mouse *Rp1* gene using recombination-based gene-targeting techniques (Figure 2A) [44], [45]. The neomycin selection cassette was then removed by crossing F1 *Rp1*-Q662X-Neo mice with universal FLPe deleter mice to generate the final excised targeted *Rp1*-Q662X knock-in allele (ki) (Figure 2A) [46]. Intercrosses of heterozygous *Rp1* ^+/Q662X^ mice generated the expected number of wild-type, *Rp1* ^+/Q662X^ and *Rp1* ^Q662X/Q662X^ mice. The mutant mice are fertile, have normal weights and a normal lifespan.

To determine if the mutant *Rp1*-Q662X mRNA is spliced correctly and produces a truncated protein in photoreceptor cells, retinas from 4-week-old *Rp1* ^+/+^, *Rp1* ^+/Q662X^, and *Rp1* ^Q662X/Q662X^ mice were evaluated using RT-PCR, Western blot and immunofluorescence analyses. The RT-PCR results showed that mutant *Rp1*-Q662X allele was correctly spliced (Figure 2E). To distinguish between the wild-type Rp1 and mutant Rp1-Q662X protein, we used two anti-Rp1 antibodies: anti-N-Rp1 and anti-C-Rp1, which recognize the N-terminal portion and C-terminal portion of Rp1 respectively (Figure 2F) [35], [38]. As expected, a 240 kDa wild-type Rp1 protein was detected in the retinas from wild-type and heterozygous mice by anti-C-Rp1 and anti-N-Rp1 antibodies (Figure 2G). No full length Rp1 protein could be detected in the homozygous *Rp1* ^Q662X/Q662X^ mice by anti-C-Rp1 or anti-N-Rp1 antibodies. The levels of the normal Rp1 protein in heterozygous mice were approximately half of that detected in the wild-type mice. Probing with anti-N-Rp1 antibody revealed that a ∼74 kDa truncated protein was expressed in the heterozygous and homozygous mutant mice (Figure 2G). Interestingly, the truncated Rp1-Q662X protein was localized to the axoneme of PSCs using the anti-N-Rp1 antibody, which is identical to the location of the wild-type Rp1 protein detected by both anti-N-Rp1 and anti-C-Rp1 antibodies (Figure 2H).

**Figure 3:**
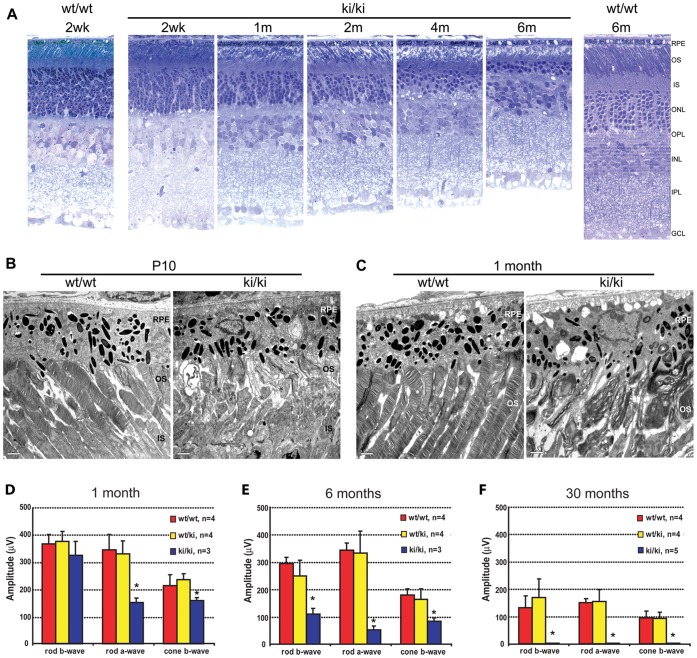
Retinal degeneration phenotype in the Rp1-Q662X mice. A. Light microscopic images of semi-thin sections of retinas from wild-type (wt/wt) and homozygous *Rp1*-Q662X knock-in mice (ki/ki) of the ages indicated. Note that thinning of the outer nuclear layer in the ki/ki mice is evident at 1 month, and progresses so that by 6 months of age only 2–3 rows of photoreceptor nuclei remain. Note also the disorganization and shortening of the photoreceptor outer segments in the ki/ki mice. (GCL, ganglion cell layer; INL, inner nuclear layer; IPL, inner plexiform layer; IS, inner segment; ONL, outer nuclear layer; OPL, outer plexiform layer; OS, outer segment; RPE, retinal pigment epithelium; 400× magnification for all images). **B, C.** Electron micrographs of retinas from wt/wt and ki/ki mice at 10 days (B) and 1 month of age (C). At 10 days of age small packets of enlarged disc membranes parallel to the axis of the axonemes replaced the normal perpendicularly orientated stacks of discs observed in control animals. These defects of outer segments were even more evident at 1 month of age, with disorganized membranous whirls in place of outer segments, compared to the well aligned, uniform discs in the control retina (IS, inner segments; OS, outer segments; RPE, retinal pigment epithelium; bars  = 2 µm). **D–F.** Average rod and cone ERG amplitudes for wild-type (wt/wt), heterozygous (wt/ki) and homozygous (ki/ki) *Rp1*-Q662X knock-in mice. The amplitudes + SD are shown for mice of the three genotypes at 1 month (D), 6 months (E) and 30 months of age (F). Significant differences (P<0.05) indicated by *. Note that the rod-a waves of the ki/ki mice were significantly decreased at 1 month of age. By 6 months of age, all measures of rod and cone function were decreased in the ki/ki mice. By 30 months of age, no recordable ERG responses were detected.

The retinal morphology and function of the mutant *Rp1* knock-in mice and their littermate controls were evaluated at indicated time points (Figure 3). Mice homozygous for the *Rp1-*Q662X allele experienced progressive retinal degeneration. At 1 month of age, the outer nuclear layer (ONL) of retinas from the *Rp1* ^Q662X/Q662X^ mice was two to three rows of nuclei thinner than that in wild-type controls. By 6 month of age, only 3–4 rows of photoreceptor nuclei remained in the *Rp1* ^Q662X/Q662X^ mice (Figure 3A). Photoreceptor cell loss was completed by 12 month of age (data not shown). Ultrastructural analyses revealed grossly abnormal outer segments in homozygous *Rp1* ^Q662X/Q662X^ mice. At 10 days of age, small packets of enlarged discs replaced the normal organized stacks of discs observed in control animals (Figure 3B). These changes were even more evident at 1 month of age, and recapitulate the phenotype observed in mice with other mutant *Rp1* alleles (Figure 3C) [36], [37]. Consistent with the structural abnormalities, the *Rp1* ^Q662X/Q662X^ mice demonstrated progressive degeneration of photoreceptor function. ERG analysis at 1 month of age showed the rod a-wave reduced by ∼50% in *Rp1* ^Q662X/Q662X^ mice compared to littermate controls (Figure 3D). By 6 months of age, all measures of rod and cone function were decreased in the *Rp1* ^Q662X/Q662X^ mice (Figure 3E). At 30 months of age, no recordable ERG responses were detected (Figure 3F). Mice heterozygous for the *Rp1*-Q662X allele did not show significant retinal structural or functional abnormality up to 30 months of age, consistent with results of our prior studies of the *Rp1*-tm1EAP mice which showed that truncated Rp1 proteins produce dominant disease on an albino background, but not a pigmented background [47] (Figure 3D, E, F).

### Generation and Characterization of Rp1 Transgenic Mice

**Figure 4:**
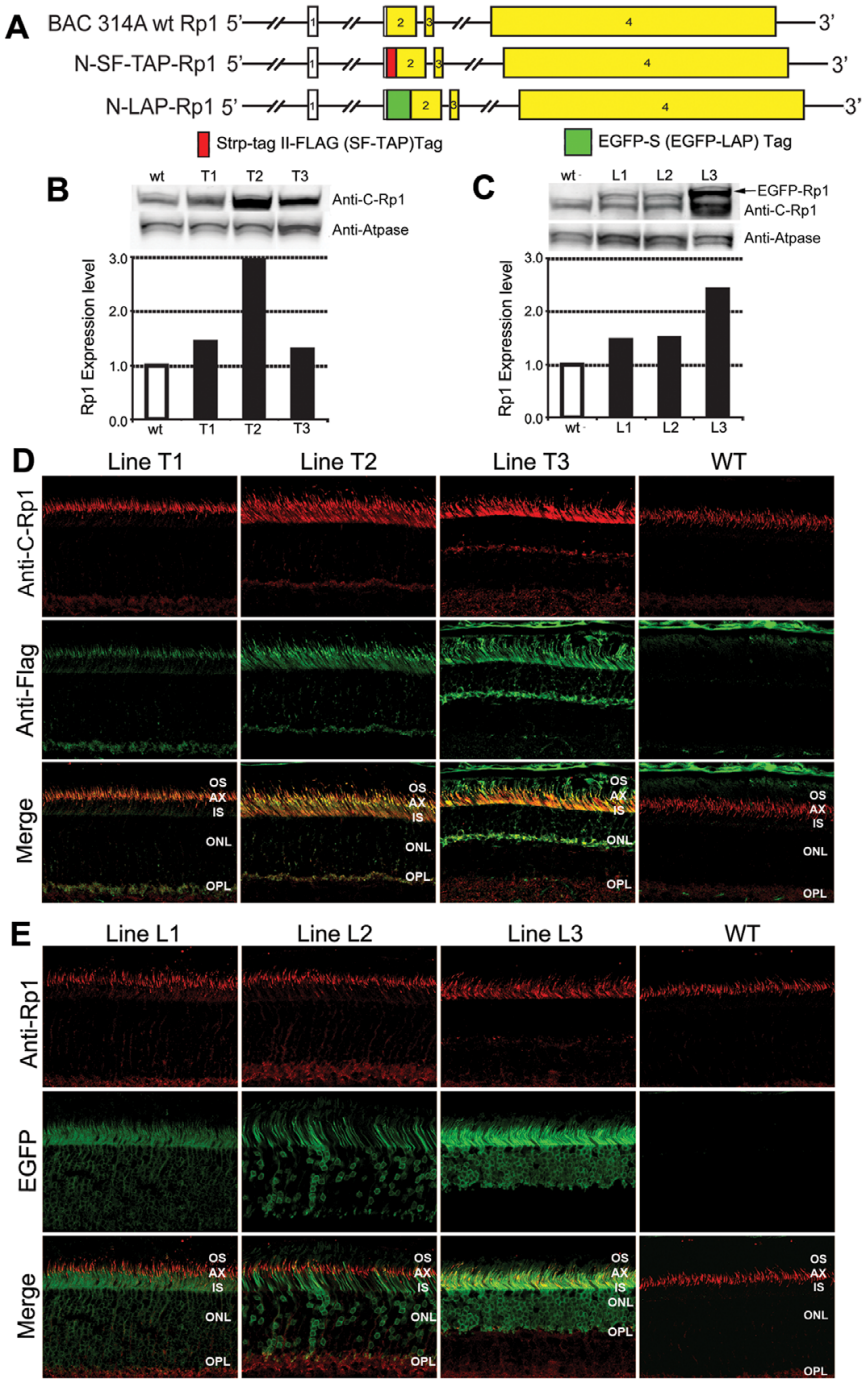
Expression of N-SF-TAP-Rp1 and N-LAP-Rp1 Transgenes. A. Diagram of *Rp1* gene, N-SF-TAP-*Rp1* and N-LAP-*Rp1* transgenes. The TAP and LAP tags were introduced into the beginning of the *Rp1* coding sequence in exon 2 in BAC 314, which contains 140 kb of mouse genomic DNA surrounding the *Rp1* locus. B-C. Western blot analyses of Rp1 proteins in N-SF-TAP-*Rp1* and N-LAP-*Rp1* mice. Equal amounts of protein from retinal extracts of wild-type, N-SF-TAP-*Rp1* and N-LAP-*Rp1* mice were analyzed by Western blotting using anti-Rp1 antibodies. The blots were also probed with antibodies to ATPase as a loading control. The Rp1 levels for the different transgenic lines were quantified and normalized to the ATPase signals. B. N-SF-TAP-*Rp1* mice. The total level of Rp1 protein in line T1 was 144% of that observed in non-transgenic littermate controls, indicating that the transgene increased expression approximately 44%, or nearly the amount expected from a third *Rp1* allele. The N-SF-TAP-*Rp1* transgene in line T2 is over-expressed relative to the wild-type protein, as it increased the total Rp1 protein level to ∼300% of normal. The total level of Rp1 protein in line T3 was only slightly elevated, but the retinas in these mice were also significantly degenerated, with 40% of the photoreceptor nuclei remaining in the outer nuclear layer, suggesting that the N-SF-TAP-Rp1 protein in this transgenic line is also 2–3 fold greater than wild-type. C. N-LAP-*Rp1* mice. The levels of N-LAP-*Rp1* fusion protein in lines L1 and L2 mice were approximately half of that observed for the wild-type Rp1 protein, again indicating that the transgene increased expression approximately the amount expected from a third *Rp1* allele. In contrast, N-LAP-*Rp1* transgene in line L3 is over-expressed, and increased the total Rp1 protein level to ∼250% of normal. D. Immunofluorescence analyses of wild-type Rp1 protein (anti-Rp1 antibodies; red) and N-SF-TAP-Rp1 protein (anti-FLAG antibodies; green) in three N-SF-TAP-*Rp1* transgenic lines and wild-type littermate control. Note that the wild-type Rp1 protein is located in the axoneme of photoreceptor outer segments. The N-SF-TAP-Rp1 protein in transgene line T1 shares the same location, as indicated by the overlap of the two signals in the merged image panel (bottom left). There is also some N-SF-TAP-Rp1 signal in the synaptic region of photoreceptor cells that is not present in wild-type retinas. The N-SF-TAP-*Rp1* transgenes in T2 and T3 are over-expressed relative to the wild-type protein. The over-expressed N-SF-TAP-Rp1 protein localizes correctly to PSC axonemes, but also mis-localizes to photoreceptor inner segments. The N-SF-TAP-Rp1 signal in the synaptic region is also increased, especially in the line T3 retinas. Note that the outer nuclear layer is thinner in the line T3 sample, consistent with the photoreceptor degeneration observed in this transgene line. The ONL is also slightly thinner in the line T2 samples as well. E. Immunofluorescence analyses of wild-type Rp1 protein (anti-Rp1 antibodies; red) and N-LAP-Rp1 protein (EGFP; green) in three N-LAP-*Rp1* transgenic lines and wild-type littermate control. The N-LAP-Rp1 protein in transgene line L1 is located in the axoneme of PSCs, like the wild-type protein. In addition, there is EGFP signal from N-LAP-Rp1 protein in the inner segments and cell bodies of the photoreceptors. Since this was not detected by the anti-Rp1 antibodies, it must be due to truncated versions of the N-LAP-Rp1 protein that retain the N-terminal EGFP tag, but have lost the C-terminal antibody binding domain. The N-LAP-*Rp1* transgenes in line L3 is over-expressed relative to the wild-type protein. The over-expressed N-SF-TAP-Rp1 protein localizes correctly to PSC axonemes, but also mis-localizes to photoreceptor inner segments and cell bodies. As for the N-SF-TAP-Rp1 line T3, there is photoreceptor degeneration in the L3 line. The N-LAP-Rp1 transgene expression in line L2 is not completely uniform, with some cells that do not express the transgene evident. In addition, there are red signals in OPL in line L1 and L2, which could represent the non-specific signaling from the anti-c-Rp1 antibody. It is also possible that this immunoreactivity of Rp1 could correspond to the C-terminal fragments of Rp1. (IS, inner segment; ONL, outer nuclear layer; OPL, outer plexiform layer; OS, outer segment; 400X magnification for all images).

Expression of correctly localized truncated Rp1 protein in the retinas of the *Rp1*-Q662X mice raised the possibility that the truncated protein could have acquired an altered function, which disrupts the normal function of wild-type Rp1, and causes disease via a gain-of-function mechanism. However, the lack of retinal phenotype in the *Rp1* ^+/Q662X^ heterozygous mice on the mixed 129Sv/C57BL6 genetic background used in the present studies did not allow us to formally distinguish between different disease mechanisms in heterozygous mice. As an alternative approach to address this question, we generated transgenic mice that express epitope-tagged versions of the full-length Rp1 protein, and then transferred the wild-type *Rp1* transgene onto the *Rp1^Q662X/Q662X^* background to study the interaction of mutant and wild-type Rp1 protein, and determine if the addition of wild-type Rp1 protein can modulate the phenotype in *Rp1^Q662X/Q662X^* mutant mice. For these studies, we placed the SF-TAP (Strep-tag II - FLAG) and LAP (EGFP - S-tag) tags at the N-terminus of the *Rp1* coding sequence in a BAC that contains 140 kb of genomic DNA surrounding *Rp1* (Figure 4A) [48], [49]. Three lines of N-SF-TAP-*Rp1* transgenic mice (designated T1–T3) and three lines of N-LAP-*Rp1* transgenic mice (designated L1–L3) were produced following microinjection of each BAC into oocytes [50]. When backcrossed with C57BL/6J mice, all six founders passed the N-SF-TAP-*Rp1* or N-LAP-*Rp1* transgenes to approximately half of their offspring.

To determine the expression level and location of the tagged Rp1 proteins produced by the N-SF-TAP-*Rp1* and N-LAP-*Rp1* transgenes, retinas from 2-month-old T1–T3 and L1–L3 transgenic lines were evaluated using Western blot and immunofluorescence analyses (Figure 4B–4E). In the N-SF-TAP-*Rp1* transgenic lines, the N-SF-TAP-Rp1 protein was expressed at a normal level in line T1, but over-expressed in lines T2 and T3 (Figure 4B). The total level of Rp1 protein in line T1 was 144% of that observed in non-transgenic littermate controls, indicating that the transgene increased expression approximately 44%, or nearly the amount expected from a third *Rp1* allele (Figure 4B). The N-SF-TAP-*Rp1* transgene in line T2 increased the total Rp1 protein level to ∼300% of normal. The total level of Rp1 protein in line T3 was only slightly elevated, but the retinas in these mice were also significantly degenerated, with 40% of the photoreceptor nuclei remaining in the outer nuclear layer. Providing that the expression level of tagged Rp1 proteins are proportionate to the thickness of the outer nuclear layer of photoreceptor nuclei, the N-SF-TAP-Rp1 protein level in line T3 is about 2–3 fold greater than wild-type (Figure 4B, D). The higher expression level of tagged RP1 in line T3 was also evidenced by the signal intensity of Rp1 immunostaining in the retinal sections (Figure 4D). Immunofluorescence analyses using anti-Rp1 antibody to detect the total Rp1 protein and anti-flag antibody to detect the SF-TAP tagged Rp1 protein showed that the N-SF-TAP-Rp1 protein in line T1 co-localizes with the wild-type protein in the PSC axoneme (Figure 4D). There is also some N-SF-TAP-Rp1 signal in the synaptic region of photoreceptor cells that is not present in wild-type retinas. The over-expressed N-SF-TAP-Rp1 proteins in line T2 and T3 localized correctly to PSC axonemes, but also mis-localized to photoreceptor inner segments and synaptic region, especially in the line T3 retinas (Figure 4D).

In the N-LAP-*Rp1* transgenic lines L1–L3, the N-LAP-Rp1 protein was expressed at normal levels in lines L1 and L2, but over-expressed in line L3 (Figure 4C). For all lines, the N-LAP-Rp1 protein co-localized in the PSC axoneme with the wild-type Rp1 protein. As for the N-SF-TAP-Rp1 protein, there is also some N-LAP-Rp1 signal in the synaptic region of photoreceptor cells that is not present in wild-type retinas. In addition, there were EGFP signals in the inner segments and cell bodies of the photoreceptors (Figure 4E). Since this was not detected by the anti-Rp1 antibodies, it may be due to truncated versions of the N-LAP-Rp1 protein that retain the N-terminal EGFP tag, but have lost the C-terminal antibody binding domain. The N-LAP-*Rp1* transgene expression in line L2 is not completely uniform, with a mixture of cells that do and do not express the transgene. As for the T3 line, there is also photoreceptor degeneration in the L3 line, with fewer photoreceptor nuclei remaining in the outer nuclear layer.

**Figure 5:**
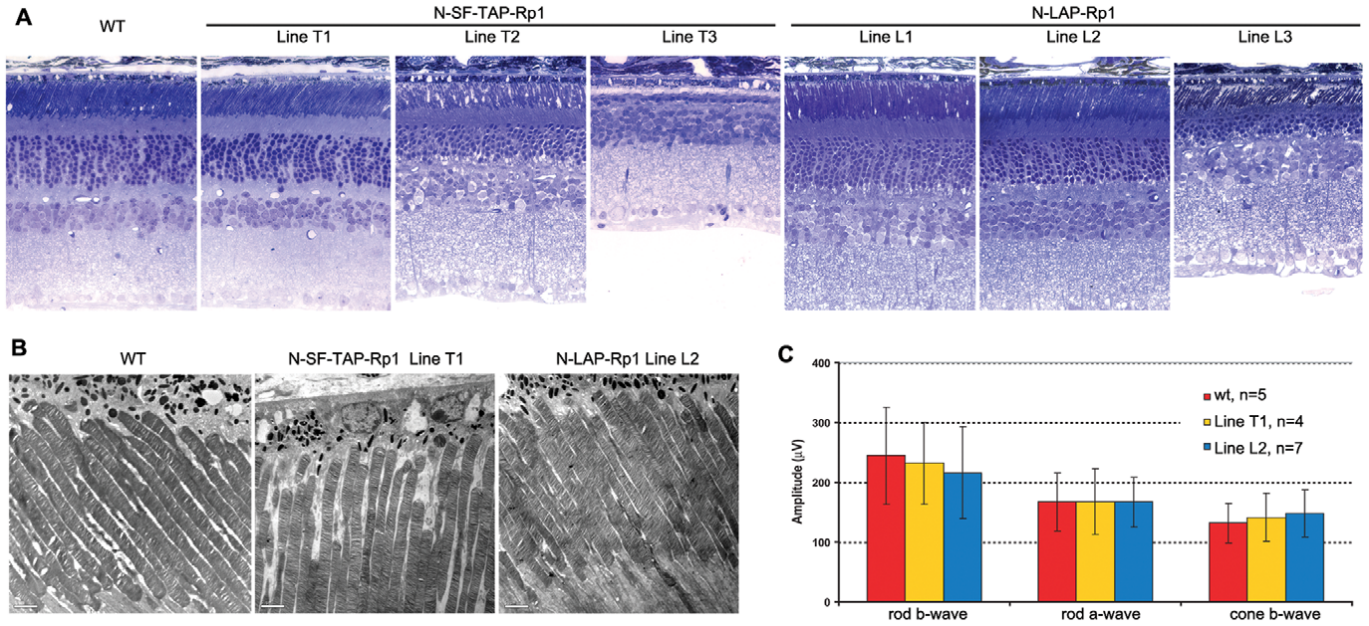
Retinal phenotypes of N-SF-TAP-Rp1 and N-LAP-Rp1 transgenic mice. **A.** Retinal histology from 1-year-old mice of the genotypes indicated. Note that the retinal structure of the N-SF-TAP-*Rp1* line T1 and N-LAP-*Rp1* lines L1 and L2 is normal. In contrast, there is photoreceptor degeneration evident in N-SF-TAP-*Rp1* lines T2 and T3, and N-LAP-*Rp1* line L3, with loss of photoreceptor nuclei and shortening of photoreceptor outer segments. (INL, inner nuclear layer; IS, inner segment; ONL, outer nuclear layer; OS, outer segment; 400× magnification for all images). **B.** Ultrastructure of photoreceptor sensory cilia in one-year old N-SF-TAP-*Rp1* line T1 and N-LAP-*Rp1* line L2 mice, compared to that of wild-type littermate control. Note that the structure of the PSC and organization of the outer segment discs are normal in both transgenic lines, consistent with the histology shown in A. **C.** Amplitudes of ERG responses from 1-year-old N-SF-TAP-*Rp1* line T1 and N-LAP-*Rp1* line L2 mice, compared to that of wild-type littermate controls. Note that the rod and cone ERG amplitudes are normal in both lines of transgenic mice.

To further evaluate the long-term effects of different levels of additional wild-type Rp1 protein on retinal function, detailed retinal histologic and functional analyses were performed in these transgenic lines at 1 year of age (Figure 5). Results showed that the histology of photoreceptors in lines T1, L1 and L2 are well preserved in mice up to 1 year of age (Figure 5A). In contrast, lines T2, T3 and L3 that over-express the tagged transgenes showed photoreceptor degeneration at 1 year of age, with shortened PSCs and loss of photoreceptor nuclei. Ultrastructural analyses of Line T1 and L2 confirmed normal PSC structure in these two lines of transgenic mice (Figure 5B). Consistent with the normal histology, ERG analysis showed that line T1 and L2 mice have normal retinal function at 1 year of age (Figure 5C). All together, these data suggest the N-SF-TAP-Rp1 and N-LAP-Rp1 proteins localize correctly to the PSC axonemes and function normally, but also indicate that over-expression of the transgene is associated with mislocalization of the tagged proteins and can lead to photoreceptor degeneration.

### Expression of Additional Full-length Rp1 Protein can Delay Photoreceptor Degeneration in Rp1^Q662X/Q662X ^Mice

The classic method to distinguish between dominant-negative and gain-of-function mechanisms of a dominant mutation is to evaluate the effect of different ratios of wild-type to mutant protein on modulating the phenotype [9], [10]. This can be achieved by creating transgenic mice expressing either a mutant allele on a wild-type background, or a wild-type allele on a mutant background. In this study, we transferred the wild-type N-SF-TAP-*Rp1* or N-LAP-*Rp1* transgenes onto the homozygous *Rp1*-Q662X background to generate new transgenic lines. Animals carrying two mutant Q662X alleles at the endogenous *Rp1* locus and a tagged *Rp1* transgene were designated as *Rp1* ^Q662X/Q662X^ : N-SF-TAP-*Rp1* or *Rp1* ^Q662X/Q662X^ : N-LAP-*Rp1* mice. We used lines T1 and L2 for these experiments, since these lines express the tagged-Rp1 proteins at relatively normal levels, and do not exhibit photoreceptor degeneration.

**Figure 6:**
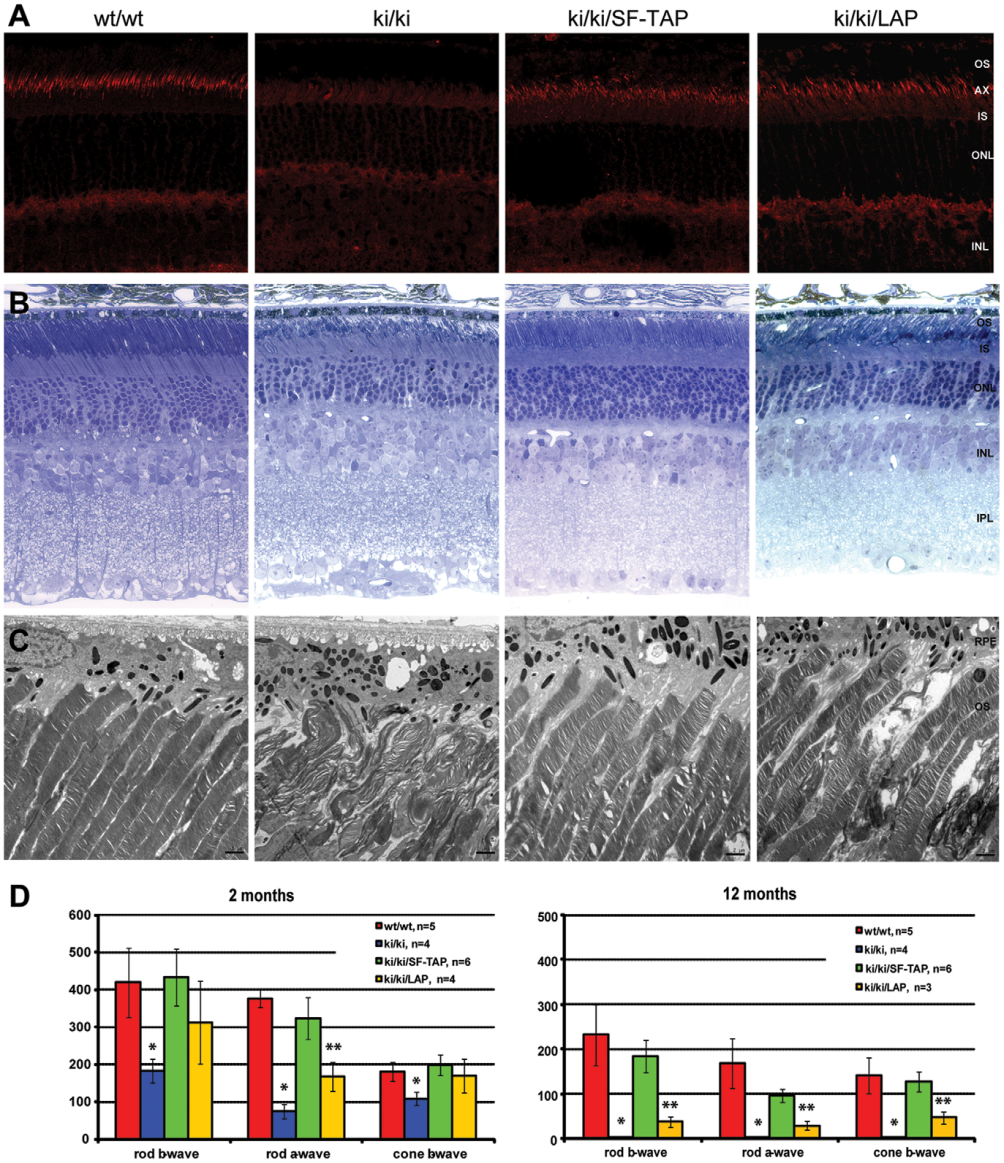
Expression of the full-length N-SF-TAP-Rp1 and N-LAP-Rp1 proteins prevents photoreceptor degeneration in Rp1^Q662X/Q662X^ knock-in mice. A. Frozen sections of retina from 2-month-old mice of the genotypes indicated were stained with anti-C-Rp1 antibodies (red). The wild-type Rp1 protein is located in the axonemes of PSC; full-length Rp1 protein is not detected in the *Rp1*-Q662X knock-in mice. Full-length Rp1 protein is also detected in the PSC axonemes of the *Rp1*-Q662X : N-SF-TAP-*Rp1* and *Rp1*-Q662X : N-LAP-*Rp1* mice. There is a mosaic pattern of full-length Rp1 protein expression in the *Rp1*-Q662X : N-LAP-*Rp1* mice. (INL, inner nuclear layer; IS, inner segment; ONL, outer nuclear layer; OS, outer segment; 400X magnification for all images). B. Retinal histology from 2-month-old mice of the genotypes indicated. The retinal structure of the *Rp1*-Q662X : N-SF-TAP-*Rp1* mice is normal, in contrast to the early photoreceptor degeneration in the *Rp1*-Q662X mice. There is partial preservation of retina structure in the *Rp1*-Q662X : N-LAP-*Rp1* mice (INL, inner nuclear layer; IS, inner segment; ONL, outer nuclear layer; OS, outer segment; 400× magnification for all images). C. Ultrastructure of PSCs in two-month old mice of the genotypes indicated. Note that the structure of the PSC and organization of the outer segment discs are normal in the *Rp1*-Q662X : N-SF-TAP-*Rp1* mice, in contrast to the disorganized PSC observed in the *Rp1* ^Q662X/Q662X^ mice. There is a mixture of cells with normal PSC and cells with disorganized PSC in the *Rp1*-Q662X : N-LAP-*Rp1* mice (OS, outer segment; RPE, retinal pigment epithelium. Bars  = 2 µm). D. Amplitudes of ERG responses from 2 month-old and 12-month-old mice of the genotypes indicated. At 2 months not that the rod and cone ERG amplitudes are close to normal in the *Rp1*-Q662X : N-SF-TAP-*Rp1* mice, in contrast to the reduction of photoreceptor function observed in the *Rp1* ^Q662X/Q662X^ mice. There is partial restoration of photoreceptor function in the *Rp1*-Q662X : N-LAP-*Rp1* mice. At 12 months photoreceptor function is relatively well preserved in the *Rp1*-Q662X : N-SF-TAP-*Rp1* mice. There is also some residual photoreceptor function in the *Rp1*-Q662X : N-LAP-*Rp1* mice. Significant differences compared to controls are indicated by * (P<0.01) and ** (P<0.05).

As described above, the *Rp1* ^Q662X/Q662X^ mice do not express full-length Rp1 protein and experience photoreceptor degeneration, with decreased retinal function evident as early as 1 month of age. Addition of the N-SF-TAP-*Rp1* and N-LAP-*Rp1* wild-type transgenes to the *Rp1* ^Q662X/Q662X^ mutant mice resulted in restoration of expression of full-length Rp1 protein in the retina. As shown in Figure 6A, the transgenic full-length Rp1 protein was localized normally to the axonemes of PSCs. There is a mosaic pattern of full-length Rp1 protein expression in the *Rp1* ^Q662X/Q662X^ : N-LAP-*Rp1* mice, which is consistent with the expression pattern of tagged Rp1 in line L2.

The retinal structure was almost completely preserved in the *Rp1* ^Q662X/Q662X^ : N-SF-TAP-*Rp1* mice at 2 months of age. In the *Rp1* ^Q662X/Q662X^ : N-LAP-*Rp1* mice, electron microscopy showed a mixture of PSCs with normal and aberrant structures (Figures 4E, 6C). At the age of 12 months, there was notable photoreceptor degeneration observed in the *Rp1* ^Q662X/Q662X^ mice, with only a single layer of photoreceptor cells with no visible outer segments remaining (Figure S1). In contrast, the photoreceptor cells and organization of outer segments was greatly preserved in the *Rp1* ^Q662X/Q662X^ : N-SF-TAP-*Rp1* mice. In the *Rp1* ^Q662X/Q662X^ : N-LAP-*Rp1* mice, more significant retinal degeneration was observed, including shorter outer segments with a mixture of normal discs and disorganized discs (Figure S1). The phenotype seen in the *Rp1* ^Q662X/Q662X^ : N-LAP-*Rp1* mice is consistent with the mosaic pattern of expression of the N-LAP-*Rp1* transgene in the line L2 mice (Figures 4E, 6C). These data also indicate that Rp1 acts in individual cells in a cell-autonomous fashion, consistent with its function as a photoreceptor MAP [38].

Consistent with the improved retinal structure, *Rp1* ^Q662X/Q662X^ : N-SF-TAP-*Rp1* mice had significantly improved scotopic and photopic ERG responses compared to *Rp1* ^Q662X/Q662X^ mice (Figure 6D). At 2 months of age, the rod a-wave, rod b-wave and cone b-wave in the *Rp1* ^Q662X/Q662X^ : N-SF-TAP-*Rp1* mice showed no significant difference compared to the wild type controls. At the age of 12 months, when there were no detectable ERG responses in the *Rp1* ^Q662X/Q662X^ mice, the maximum amplitude of the rod a-wave, rod b-wave and cone b-wave were preserved to 57%, 71% and 90% of normal levels, respectively, in the *Rp1* ^Q662X/Q662X^ : N-SF-TAP-*Rp1* mice; these values were not statistically different from those observed in the control mice. The restoration of retinal function in the *Rp1* ^Q662X/Q662X^ : N-LAP-*Rp1* mice was not as complete as that in *Rp1* ^Q662X/Q662X^ : N-SF-TAP-*Rp1* mice, presumably because only a portion of the photoreceptor cells in these mice expressed the N-LAP-Rp1 protein. At 2 month of age, the maximum amplitude of rod a-wave improved to 50% of normal, compared to 19% of normal in *Rp1* ^Q662X/Q662X^ mice. At 12 months of age, the maximum amplitude of rod a-wave was 17% of normal in the transgenic mice, which is similar to the level in 2-month-old knock-in mice.

## Discussion

The success of the Phase I clinical trials of gene therapy for LCA has led to rapid progress in developing genetic therapeutic strategies. However, efforts to develop gene therapy for dominant diseases have lagged behind those directed at recessive disease caused by loss-of-function mutations, in part because of the assumption that successful treatment of dominant diseases will require suppression or removal of the protein produced by the mutant allele [51]–[54]. Some successes at this suppression or removal in conjunction with replacement approach have been reported, yet this approach is clearly more complicated than gene augmentation therapy and it is only necessary for gain-of-function mutations [9], [10]. Hence, it is critical to distinguish between the gain-of-function, dominant-negative and haploinsufficiency mutations when gene therapy is applied to dominant diseases. In this study, we present several important insights into the mechanisms by which mutations in *RP1* cause retinal degeneration. Data from the family with a frameshift mutation in exon 3 of *RP1* confirm that null alleles of *RP1* cause arRP, but haploinsufficiency of *RP1* does not cause RP. Data from the *Rp1*-Q662X knock-in mice show that mutant alleles with truncating mutations in the final exon can produce truncated proteins that retain their microtubule binding domains and localize correctly to the axonemes of PSCs. Photoreceptor degeneration in the *Rp1*-Q662X mice can be delayed or prevented by increasing the ratio of wild-type to mutant protein without removal of the mutant protein, demonstrating that the truncated Rp1-Q662X protein expressed in these mutant mice does not exert a toxic gain-of-function in the retina [9], [10]. These findings are important because they indicate that gene augmentation therapy, such as that used for recently reported LCA clinical trials, has the potential to be beneficial for both dominant and recessive *RP1* patients. It is also evident that the levels of protein delivered for therapy will have to be carefully controlled because over-expression of Rp1 causes retinal degeneration. These results also raise the question of how many other forms of dominant retinal disorders have a comparable etiology, and thus could also be amenable to gene augmentation therapy.

The human genetic study of the family with the p.P229GfsX35 mutation in exon 3 of *RP1* provides confirming evidence that haploinsufficiency of *RP1* cannot cause dominant RP. Because the premature termination codon produced by the resulting frameshift occurs in exon 3, the transcripts with the identified 1-bp deletion are expected to be subject to NMD, resulting in no expression of the mutant RP1 protein [43]. Direct evidence of NMD of this mutant *RP1* allele in the retina cannot be obtained from the patient; further study using lymphoblasts derived from the patient and/or an animal models is warranted. The parents and sister of the affected patient are heterozygous for this frameshift mutation, but show no evidence of retinal degeneration. This result is consistent with the recent report by Chen *et al*, who described a patient with recessive RP due to two novel frameshift mutations in *RP1*, and family member carrying a S2RfsX16 mutation in exon 2 that is unaffected [41]. Together, these findings support the idea that functional hemizygosity of *RP1* does not cause disease.

The *Rp1*-Q662X mice provide the first clear demonstration that mutant *Rp1* mRNA with a premature stop codon in the final exon can escape NMD, and produce truncated protein in photoreceptor cells *in vivo*. We have previously generated a gene targeted *Rp1*-tm1EAP mouse model that carries an artificial stop codon in last exon of *Rp1* gene [37], [47]. In the *Rp1*-tm1EAP mice, the mutant *Rp1* allele was produced by removing the majority of exon 4, and thus did not allow for testing the hypothesis that the premature termination codons in last exon lead to the production of truncated Rp1 proteins. The *Rp1*-Q662X allele produces the same 74 kDa truncated Rp1 protein localized correctly to the axonemes of PSCs as that produced in the retinas of *Rp1*-tm1EAP mice, consistent with retention of the DCX microtubule binding domains (amino acids 35–236) in the truncated N-terminal Rp1 protein. These results are also consistent with those from PCR of illegitimate transcripts from lymphoblasts of patients with the R677X mutation in *RP1* [37]. It is hypothesized that in patients with truncating *RP1* mutations in the last exon, the mutant truncated protein is expressed and retains its ability to binding axonemal microtubules in the photoreceptor cells.

The *Rp1*-Q662X mutant allele causes disorganized outer segment discs, but only when present in the homozygous state in the study reported here. The lack of phenotype in *Rp1* ^+/Q662X^ heterozygous mice recapitulates the observation in *Rp1*-tm1EAP mice that a single *Rp1* mutant allele in *Rp1*-tm1Eap mice causes dominant disease only on an congenic albino (A/J) background, but not on pigmented (mixed 129Sv/C57BL/6J) background [47]. Since the truncated Rp1 proteins produced by the *Rp1*-tm1EAP and *Rp1*-Q662X mouse models are the same, we expect that the truncated Rp1-Q662X is functional in the retina and a single copy of Rp1-Q662X would cause dominant disease when it is expressed on an albino background. Genetic factors are also thought to play a role in modulating *RP1* disease expression in human patients [26], [27], [47]. While most truncating mutations in exon 4 of *RP1* cause adRP, it has been reported that truncating mutations in *RP1* may cause recessive disease in several Indian and Pakistani families (Figure 1A) [34], [40]. We hypothesize that the mode of inheritance and/or the severity of disease caused by truncating mutation in *RP1* or *Rp1* may be controlled is part by genetic background or modifier genes. Evidence in support of this hypothesis includes the observation that the truncated protein produced by the *Rp1*-tm1EAP mice causes dominant disease on an albino background, but not pigmented backgrounds. The variation in *RP1* disease severity may also be explained by sequence variants in genes that encode proteins that interact with RP1, or the level of expression of the normal allele, and warrants additional investigation [55], [56]. Additionally, the stability and localization of different mutant RP1 proteins produced in photoreceptor cells may contribute to determining the severity of RP1 disease. It has been experimentally demonstrated that the relative degree of protein retention/degradation and the subcellular localization of mutant proteins produced by different truncating mutations at a single site in the *ROR2* gene can determine the phenotypic outcome [57].

The data from several lines of *Rp1* transgenic mice showed that the phenotype of the transgenic mice and the location of the protein expressed by additional transgene are closely correlated to the protein expression levels in retina. In the N-SF-TAP-*Rp1* line T1 and N-LAP-*Rp1* line L2 mice that produce Rp1 proteins in amounts similar to those produced from a single endogenous *Rp1* allele, the tagged Rp1 proteins were located normally in the axonemes of PSCs, and the retinal structure and function was normal up to at least one year of age in these transgenic line. This suggests that the epitope tags do not interfere with Rp1 function and the expression of 50% more Rp1 protein than usual is well-tolerated by photoreceptor cells. The tagged Rp1 proteins were partially mis-localized to other subcellular locations when expression of these proteins exceeded normal levels. Over-expression of *Rp1* also resulted in photoreceptor degeneration, as has been reported for several other photoreceptor proteins, such as rhodopsin [58], [59]. These findings imply that the levels of Rp1 protein are tightly regulated and thus, the levels of protein delivered for therapy, such as by gene therapy, will have to be carefully controlled. In this study, we found that a ratio of 1∶2 of wild-type vs. mutant protein is enough to significantly reduce the retinal degeneration caused by the truncated *Rp1* protein, as evidenced by the relative preservation of retinal function and structure in the 12-month-old *Rp1* ^Q662X/Q662X^ : N-SF-TAP-*Rp1* mice compared to the *Rp1* ^Q662X/Q662X^ mice. The level of full-length RP1 protein required to rescue the structure and function of photoreceptor cells in heterozygous mice or patients will need to be determined.

It is worth noting that in the N-LAP-*Rp1* transgenic mice, EGFP signal was found separately from Rp1 signal detected by anti-C-terminal Rp1 antibody (Figure 4). This suggests that the EGFP-Rp1 fusion protein underwent proteolysis, resulting in separation of the LAP-tag-containing N-terminal portion of the protein from the C-terminal portion of the protein to which the antibodies used are directed [35]. This finding indicates that care is needed interpreting data obtained using EGFP and other fluorescent fusion proteins to localize proteins of interest in cultured cells and *in vivo*. While it has been suggested by other authors that validation of fluorescent fusion protein location by detecting the endogenous protein is important, we have not found other reports of separation of the EGFP tag from fusion proteins *in vivo* [60].

In this study, although the *Rp1*-Q662X mice on a mixed 129Sv/C57BL/6J genetic background are not a model of dominant disease to test gene therapeutic approaches, the homozygous *Rp1^Q662X/Q662X^* mice provide a valuable model system to determine if the truncated Q662X protein has a gain-of-function or a dominant negative effect in photoreceptor cells *in vivo* by expressing a wild-type *Rp1* allele on this mutant background. The results from the crossing of *Rp1^Q662X/Q662X^* knock-in mice and *Rp1* BAC transgenic mice showed that the phenotype of the *Rp1^Q662X/Q662X^* mice can be rescued without first removing the mutant protein, indicating that the truncated Rp1 protein does not exert a toxic gain-of-function effect in the photoreceptor cells in mutant *Rp1* animal models.

Based on the findings from human and animal studies, which rule out haploinsufficiency and gain-of-function as the cause of RP1 disease, the most logical conclusion would be that truncated RP1 proteins cause disease via a dominant-negative mechanism [9], [10]. This concept is consistent with the fact that all adRP mutations are spread over a large protein region in the last exon and that there are no missense mutations that lead to a very specific gain-of-function. It is also consistent with the findings that, in both *Rp1*-Q662X and *Rp1*-tm1EAP mouse models, truncated Rp1 proteins retain their ability to bind to axonemal microtubules in the photoreceptor cells, but may have lost the ability to interact with other proteins that are hypothesized to bind to the C-terminal portion of Rp1 and mediate organization of outer segment discs along the axoneme [37], [38]. It is hypothesized that, in dominant *RP1* disease, the truncated RP1 proteins does not cause disease by generating a novel toxic function, but rather compete for binding to axonemal microtubules with full length RP1 protein, and thus interfering with RP1-mediated organization of outer segment discs along the axoneme. Additional studies of the genetic mechanism of and the gene therapeutic approaches for dominant *RP1* disease using *Rp1*-Q662X or *Rp1*-tm1EAP mice on an albino background are warranted to further test this hypothesis.

The finding that truncating mutations in *RP1* does not cause dominant disease via a gain-of-function mechanism raises the question of how many other dominant forms of IRD are caused by a similar mechanism. Despite the great progress made in recent years discovering novel IRD disease genes, the mechanisms by which the identified mutations cause disease have only been evaluated thoroughly *in vivo* for a limited number of genes [61]–[63]. An important example of dominant IRD is RP caused by mutations in the rhodopsin (*RHO*) gene, which are the most common cause of adRP [18], [64]. It is evident that haploinsufficiency of *RHO* does not cause dominant RP, since people heterozygous for null *RHO* mutations and heterozygous *Rho* knockout mice do not develop retinal degeneration [65]. The most common mutation adRP mutation in *RHO* is the P23H mutation, which accounts for 10 to 15% of adRP in Western populations [18]. There are conflicting data, however, about whether P23H mutation in *RHO* causes adRP via a gain-of-function or dominant-negative mechanism [66]–[70]. A recent report that *RHO* gene augmentation therapy preserves retinal function in P23H *RHO* transgenic mice is consistent with a dominant-negative effect of the P23H mutation [71]. It is possible, therefore, that the P23H mutation in *RHO*, and other dominant mutations in *RHO* and other IRD disease genes cause disease via dominant-negative mechanisms, and thus like dominant *RP1* disease may also be amendable to gene augmentation therapy.

## Materials and Methods

### Clinical Evaluations

The clinical study was approved by the ethical review board of The Rotterdam Eye Hospital and conformed to the tenets of the Declaration of Helsinki. Written consent was given by the participants for their information to be stored in the hospital database and used for research. Retrospective data of the index patient were evaluated. Ophthalmic examination of all family members included best-corrected visual acuity (Snellen), slitlamp examination, and fundoscopy. Fundus autofluorescense images (30^o^, alignment of 15 frames) were obtained with a confocal scanning laser ophthalmoscope with 488 nm excitation (Spectralis®, Heidelberg Engineering, Heidelberg, Germany). Electroretinograms (according to ISCEV standards) were performed in the index patient and his parents [72]. Fundus photographs were obtained in the index patient and his father.

### Genome-wide Homozygosity Mapping

In a large study aiming at the identification of the genetic causes of RP in The Netherlands via homozygosity mapping [42], 231 Dutch arRP patients were genotyped on a high-resolution Genome-wide Human SNP array 5.0 that contains 500,000 SNPs and 420,000 non-polymorphic probes for copy number detection (Affymetrix, Santa Clara, CA, USA). Homozygous regions in the patients’ genome were calculated using Partek Genomics Suite software (Partek, St. Louis, MO, USA). Stretches of DNA that contained 250 or more homozygous SNPs (1.5 Mb on average) were considered to be significant homozygous regions.

### RP1 Sequence Analysis

Patients that were homozygous for the genomic region containing the *RP1* gene were selected for sequence analysis. Primers to amplify all exons and intron-exon boundaries of *RP1* are available on request. PCR products were sequenced with the ABI PRISM Big Dye Terminator Cycle Sequencing V2.0 Ready Reaction kit and the ABI PRISM 3730 DNA analyzer (Applied Biosystems, Foster City, CA, USA).

### Generation of Rp1^Q662X/Q662X^ Knock-in Mice

This research followed the tenets of the ARVO Statement for the Use of Animals in Ophthalmic and Vision Research, and the guidelines of the Massachusetts Eye and Ear Infirmary for Animal Care and Use, and was specifically approved by Institutional Animal Care and Use Committees at the Massachusetts Eye and Ear Infirmary. The *Rp1*-Q662X targeting vector was generated from a mouse bacterial artificial chromosome (BAC) clone that contains the entire mouse *Rp1* gene using modified recombineering techniques (Fig. 2A). This BAC clone (314) was identified by screening high-density filters from the RPCI-22 (129S6/SvEvTac) Mouse BAC Library (BACPAC web site: http://www.chori.org/bacpac/libraryres.htm/provided in the public domain by the National Center of Excellence in Genomics at Children’s Hospital Oakland Research Center, Oakland, CA). Briefly, the neomycin-zeocin resistance cassette from plasmid pKOEZ59 was inserted between exons 3 and 4 of the *Rp1* gene in BAC 314 using recombination-based techniques [45], [73]. A 23.8 kb portion DNA containing the modified *Rp1* gene was then retrieved into pBluescript II via homologous recombination, to create wild type construct [74]. This vector was mutagenized to introduce the Q662X mutation and then linearized to transfect into AB2.2 mouse ES cells (obtained from Dr. Allan Bradley at the Wellcome Trust Sanger Institute, UK website: http://www.sanger.ac.uk/resources/mouse/micer/) [75]. Approximately 250 G418 resistant colonies were selected and expanded according to established techniques [50]. DNA prepared from the isolated clones was digested with *Bam*HI and analyzed by Southern blot using a 5′ probe to identify correctly targeted ES cells. DNA from the single positive recombinant was also digested with *Pme*I, and analyzed by Southern blot using a 3′ probe. Probes for Southern blot analysis were amplified from BAC 314A DNA, and cloned into the pCRII-TOPO vector (Invitrogen, Carlsbad, CA). The 5′ probe was amplified with primers 5′-CAAGAATCTGGCGGCCGCTGGACTGAATGTCAC-3′, and 5′- GAGAATAGGAACTTCGATCCATAACTTCGTATAATG-3′, and the 3′ probe with primers 5′-GAATCCAGGTGGGGAAGAGCACGGGTAA-3′ and 5′-TATTTTATCTAAAAGACCTATCTCGAATCC-3′ (Figure 2B).

One ES cell clone with the *Rp1*-Q662X-Neo allele was injected into blastocysts to generate chimeric mice [50]. Chimeric mice were generated using standard techniques, and the Frt-flanked neomycin selection cassette was removed by crossing the *Rp1*-Neo-Q662X mice with universal FLPe deleter mice [46]. The final excised targeted *Rp1*-Q662X knock-in allele was verified by PCR and sequencing (Figure 2C). Heterozygous and homozygous animal were generated by intercrossing the F1 progeny. Genotyping was performed by PCR analysis of tail DNA using primers: 5′-TACTGTTTTTCCTTGGCTTACTC-3′ and 5′-ACTTTGGCTGTTGTGTTTCTTA-3′. The sizes of the PCR products amplified from wild-type and mutant *Rp1* alleles are 231 bp and 497 bp, respectively (Figure 2D).

### Generation of Rp1 BAC Transgenic Mice

To attempt to fully recapitulate *Rp1* expression with a transgene, we used a BAC genomic clone containing a 140 kb fragment of mouse genomic DNA to produce the transgenic mice. This 140 kb fragment encompassed the complete *Rp1* gene and, in addition, contained 53 kb of 5-flanking DNA and 62 kb of 3-flanking DNA (Fig. 4). The transgenic construct was produced by incorporating two different tags, SF-TAP (Strep-tag II-FLAG) and LAP (EGFP-S-tag) into the N-terminus of the *Rp1* coding sequence in exon 2 of the *Rp1* gene in the BAC (Fig. 4A) [48], [49]. Three lines of N-SF-TAP-*Rp1* transgenic mice (designated T1–T3) and three lines of N-LAP-*Rp1* transgenic mice (designated L1–L3) were produced following microinjection of each BAC into oocytes [50]. The *Rp1* transgenes present in the *Rp1* transgenic lines were distinguished from the endogenous *Rp1* locus by PCR amplification with TAP-specific primers 5′-CATTGTTTGAGTGTAAATATCCGCATTGG-3′, 5′-CGCTTCCTCCTCCCTTCTCGAACTGAG-3′ and LAP-specific primers 5′-TCGCCGGACACGCTGAACTTGTG-3′, and 5′-CATTGTTTGAGTGTAAATATCCGCATTGG-3′. Transgenic progeny were backcrossed to C57BL/6J mice to establish lines from each founder. In the present study, transgenic line T1 and L2 were used for crosses with *Rp1*-Q662X knock-in mice. All progeny were then genotyped for the presence of the *Rp1* transgene and for the presence of the mutant Q662X allele.

### Electroretinography

Electroretinography (ERG) testing in the mice was performed as previously described [37]. In brief, the pupils of dark-adapted, anesthetized mice were dilated with 1% tropicamide and the mice were placed on a stage maintained at 37°C. Retinal responses were detected with platinum electrodes placed in contact with the corneas. A platinum wire loop placed in the mouth served as the reference and a ground electrode. ERG were recorded with Espion Electrophysiology System (Diagnosys LLC, Lowell, MA) calibrated by the vendor. A stage with the mouse was positioned in such a way that the mouse head was located inside the ColorDome stimulator thus ensuring full-field uniform illumination. All manipulations were performed under dim red light, and after being placed into the ColorDome the animal was left in darkness for 10 min for complete dark re-adaptation. The ERG recordings followed in general guidelines recommended by the “Standard for Clinical Electroretinography” [76]. The rod b-wave (“weak flash rod response” by the Standard) was recorded at a response to green (λ = 513 nm) flash intensity of 1.08×10^−2^ scot cd s m^−2^. The saturating rod a-wave was elicited by a white flash of 500 scot cd s · m^−2^ intensity produced by a xenon lamp (“High-intensity ERG” by the Standard). The single cone ERG was elicited by a 500 scot cd s · m^−2^ intensity white flash delivered on a 30 scot cd · m^−2^ background suppressing the rod activity. The ERGs were recorded in a 0.1 to 300 Hz bandwidth, digitized at 1 kHz, and the amplitudes of the respective responses were measured from the baseline to peak for the a-wave, and from the trough to peak for the rod and cone b-waves. Each trace is the average of 3 to 10 individual records (the traces of the two eyes were also averaged during analysis). Statistical analysis was performed using Prism v3.02 software (GraphPad, San Diego, CA).

### RT-PCR

Retinas from animals of different genotypes at the desired ages were collected and homogenized in Trizol (Invitrogen, Carlsbad, CA). Total RNA was extracted from one retina and reverse transcribed using Superscript II (Invitrogen). The PCR reactions were then performed using 2 µL of first-strand cDNA to amplify the region of *RP1* transcript containing exon 2 to 3 and exon 3 to 4. The primer sets used were: forward, 5′-TCGGCCCTGGCTGAGTAGTCG-3′ (on exon 2), reverse, 5′-TTCCTGGTTTAAATGGCTCCCTTCC-3′ (on exon 3); and forward, 5′-TGCCAAGTTACCAGGAATCTC-3′ (on exon 3), reverse, 5′-CGACCGTCATCGTACCATCTTG-3′ (on exon 4).

### Western Blotting

Retinas from animals of different genotypes at the desired ages were collected and homogenized in sample buffer (NuPAGE; Invitrogen). Solubilized retinal protein samples were then processed for Western blot analysis as described [35]. Briefly, equivalent amounts of protein from each genotype were separated on 4% to 12% Tris-acetate gel (NuPage; Invitrogen) and transferred to polyvinylidene difluoride (PVDF) membrane (Millipore, Bedford, MA). The membranes were blocked with 10% nonfat dry milk in TBS-T and incubated with primary antibodies. Chicken polyclonal anti-C-Rp1 antibody and anti-N-Rp1 antibodies were used to detect the normal full length Rp1 and the mutant truncated Rp1 protein. Monoclonal anti-alpha 1 Sodium Potassium ATPase antibody from Abcam (Cat. ab7671) antibody was used as internal control to quantify the expression level of transgenic Rp1 protein. Antibody binding was detected with alkaline phosphatase conjugated or Odyssey IRDye secondary antibodies. Positive signals were visualized and quantified by Odyssey Infrared Imaging System (Li-Cor Biosciences, Lincoln, NE).

### Immunofluorescence, Light and Electron Microscopy

Eyes from *Rp1*-Q662X knock-in mice and transgenic mice were processed for immunostaining experiments as described previously [37]. Ten micron cryosections were cut and evaluated using anti-N-Rp1 and anti-C-Rp1 and anti-flag (Sigma, St. Louis, MO) antibodies. Sections were incubated overnight at 4°C with primary antibodies followed by Alexa 488- and Alexa 555-conjugated secondary antibodies (Invitrogen). Stained sections were viewed with a Zeiss LSM 510 Meta confocal microscope, and the images were processed with Zeiss Meta 510 software. Preparation of retinas for light and electron microscopy was performed as previously described [37], [38]. Semithin (0.75 µm) sections were cut and stained with alkaline toluidine blue for light microscopy, and 60- to 80-nM ultrathin sections were stained with 2% uranyl acetate and lead citrate, and photographed using a transmission electron microscope.

## Supporting Information

Figure S1 **Expression of the full-length N-SF-TAP-Rp1 and N-LAP-Rp1 proteins prevents photoreceptor degeneration in Rp1^Q662X/Q662X^ knockin mice.** **A.** Retinal histology from 12-month-old mice of the genotypes indicated. The retinal structure of the combined *Rp1*-Q662X : N-SF-TAP-*Rp1* mice is essentially normal, in contrast to the early photoreceptor degeneration in the *Rp1*-Q662X mice. There is partial preservation of retina structure in the combined *Rp1*-Q662X : N-LAP-*Rp1* mice (INL, inner nuclear layer; IS, inner segment; ONL, outer nuclear layer; OS, outer segment; 400× magnification for all images). **B.** Ultrastructure of PSCs in 12-month old mice of the genotypes indicated. Note that the structure of the PSC and organization of the outer segment discs are normal in the combined *Rp1*-Q662X : N-SF-TAP-*Rp1* mice, in contrast to the grossly disorganized PSC observed in the *Rp1* ^Q662X/Q662X^ mice. More significant disorganization of PSCs was observed in the combined *Rp1*-Q662X : N-LAP-*Rp1* mice, including shorter outer segments with a mixture of normal discs and disorganized discs (OS, outer segment; RPE, retinal pigment epithelium. Bars  = 2 µm).
(TIF)
