# Supplementary figures and images for: Expression of Wild-Type Rp1 Protein in Rp1 Knock-in Mice Rescues the Retinal Degeneration Phenotype
Source: PLoS One. 2012 Aug 21;7(8):e43251. doi: 10.1371/journal.pone.0043251 (PMC3424119; doi:10.1371/journal.pone.0043251)

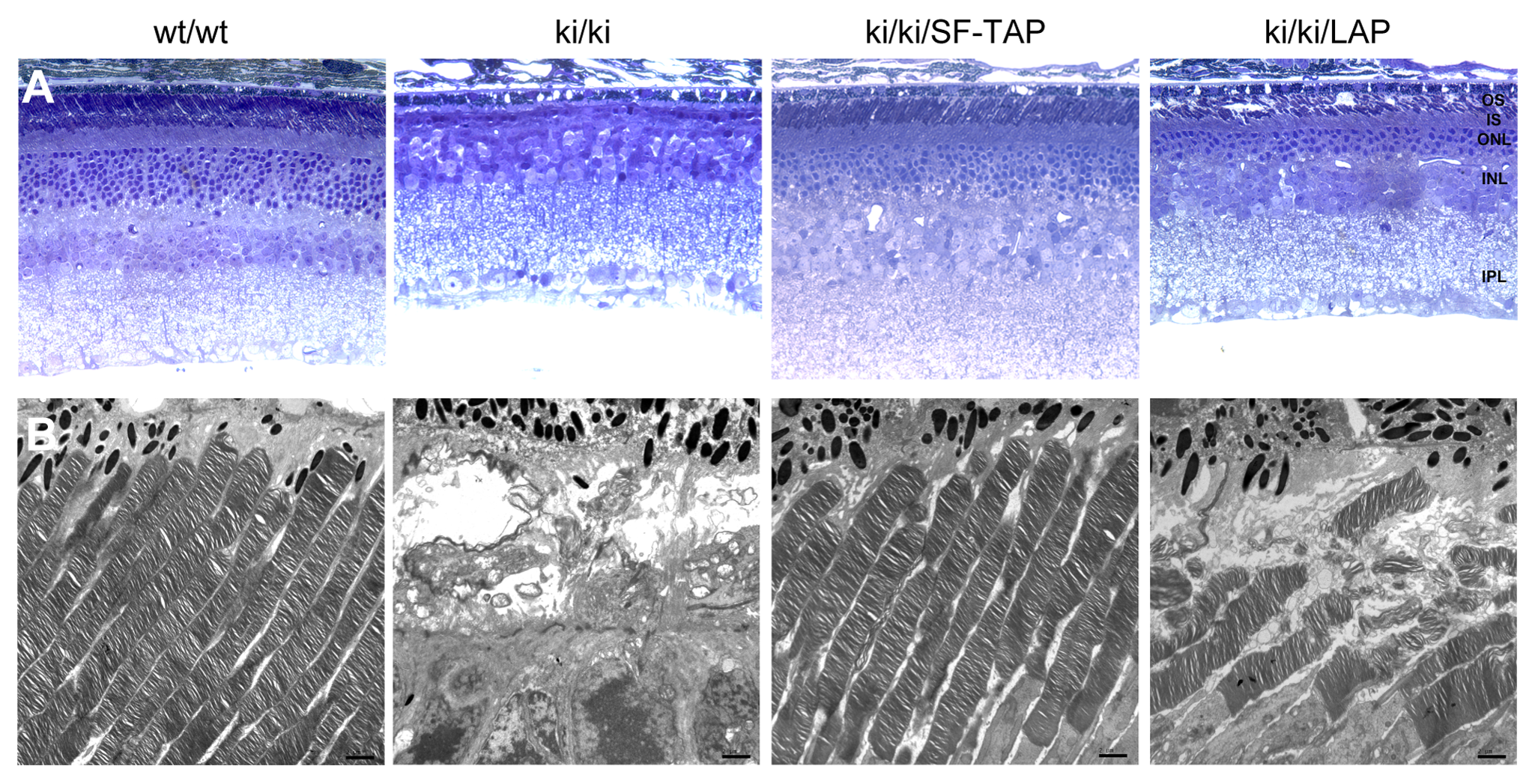

Supplement: Figure S1 — Expression of the full-length N-SF-TAP-Rp1 and N-LAP-Rp1 proteins prevents photoreceptor degeneration in Rp1Q662X/Q662X knockin mice. A. Retinal histology from 12-month-old mice of the genotypes indicated. The retinal structure of the combined Rp1-Q662X : N-SF-TAP-Rp1 mice is essentially normal, in contrast to the early photoreceptor degeneration in the Rp1-Q662X mice. There is partial preservation of retina structure in the combined Rp1-Q662X : N-LAP-Rp1 mice (INL, inner nuclear layer; IS, inner segment; ONL, outer nuclear layer; OS, outer segment; 400× magnification for all images). B. Ultrastructure of PSCs in 12-month old mice of the genotypes indicated. Note that the structure of the PSC and organization of the outer segment discs are normal in the combined Rp1-Q662X : N-SF-TAP-Rp1 mice, in contrast to the grossly disorganized PSC observed in the Rp1 Q662X/Q662X mice. More significant disorganization of PSCs was observed in the combined Rp1-Q662X : N-LAP-Rp1 mice, including shorter outer segments with a mixture of normal discs and disorganized discs (OS, outer segment; RPE, retinal pigment epithelium. Bars = 2 µm). (TIF) [file pone.0043251.s001.tif]
